# Are there any differences in clinical and laboratory findings on admission between H1N1 positive and negative patients with flu-like symptoms?

**DOI:** 10.1186/1756-0500-4-4

**Published:** 2011-01-07

**Authors:** Paul Zarogoulidis, Theodoros Constantinidis, Paschalis Steiropoulos, Nikolaos Papanas, Kostas Zarogoulidis, Efstratios Maltezos

**Affiliations:** 1 Unit of Infectious Diseases, University General Hospital of Alexandroupolis, Greece; 2 Laboratory of Hygiene and Environmental Protection, Democritus University of Thrace, Greece; 3 Lung Tumor Research Section, Aristotle University Pulmonary Clinic, Thessaloniki, Greece

## Abstract

**Background:**

The World Health Organization alert for the H1N1 influenza pandemic led to the implementation of certain measures regarding admission of patients with flu-like symptoms. All these instructions were adopted by the Greek National Health System. The aim of this study was to retrospectively examine the characteristics of all subjects admitted to the Unit of Infectious Diseases with symptoms indicating H1N1 infection, and to identify any differences between H1N1 positive or negative patients. Patients from the ED (emergency department) with flu-like symptoms (sore throat, cough, rhinorhea, or nasal congestion) and fever >37.5°C were admitted in the Unit of Infectious diseases and gave pharyngeal or nasopharyngeal swabs. Swabs were tested with real-time reverse-transcriptase-polymerase-chain-reaction (RT-PCR).

**Findings:**

Patients were divided into two groups. Group A comprised 33 H1N1 positive patients and Group B (control group) comprised of 27 H1N1 negative patients. The two groups did not differ in terms of patient age, co-morbidities, length of hospitalization, temperature elevation, hypoxemia, as well as renal and liver function. There were also no significant differences in severity on admission. C-reactive protein (CRP) (mean 12.8 vs. 5.74) and white blood count (WBC) (mean 10.528 vs. 7.114) were significantly higher in group B than in group A upon admission. Obesity was noted in 8 patients of Group A (mean 31.67) and 14 patients of Group B (mean 37.78). Body mass index (BMI) was lower in H1N1 positive than in H1N1 negative patients (mean 31.67 vs. 37.78, respectively; p = 0.009).

**Conclusions:**

The majority of patients in both groups were young male adults. CRP, WBC and BMI were higher among H1N1 negative patients. Finally, clinical course of patients in both groups was mild and uneventful.

## Background

In June 2009, the World Health Organization signaled that a novel H1N1 flu pandemic was underway [1-5]. The H1N1 virus is a triple-reassortant influenza virus containing genes from human, swine, and avian influenza viruses. This is a case control study. Patients were selected from the influenza special clinic (emergency flu department) according to their attendance. Control group were patients with flu symptoms and signs with negative serological test. Most patients with H1N1 infection present flu-like symptoms with a benign course [6]. Patients with co-morbidities may have a serious clinical presentation with hypoxemia. The main cause of death is acute respiratory distress syndrome (ARDS) [7-10]. The first case of influenza A (H1N1) virus infection in the area of Thrace, Greece was documented in the University Hospital of Alexandroupolis on 10^th ^August 2009. The purpose of this study was to investigate the potential differences on admission between H1N1 positive and negative patients with flu-like symptoms.

## Patients and Method

The University Hospital of Alexandroupolis is a report center of the H1N1 virus for the region of Thrace in Greece. The hospital consists of more than fifty departments, one of which is the Unit of Infectious Diseases. During the influenza epidemic a special department for flu was established in which all patients with flu symptoms and/or signs were referred. Patients with positive flu test were transferred in an 8 bed unit with negative pressure especially designed to quarantine and isolate patients with airborne transmitted viral infections.

From 10th August until 31st December 2009, 33 cases of confirmed H1N1 influenza A virus were hospitalized and quarantined in the Unit of Infectious Diseases. All patients with flu-like symptoms (sore throat, cough, rhino rhea, or nasal congestion) and fever >37.5°C were admitted in the Unit of Infectious diseases and gave pharyngeal or nasopharyngeal swabs. The swabs were tested with real-time reverse-transcriptase-polymerase-chain-reaction (RT-PCR) as in previous reported studies [11-13]. It should also be mentioned that although RT-PCR is the most sensitive and specific test for the diagnosis of influenza virus infection, upper respiratory tract specimens are not as specific (~80%) as lower respiratory tract specimens (~100%) [14]. All results were given in a period of time from 8 to 48 hours, and all patients remained under quarantine and isolation in negative pressure chambers according to WHO guidelines [15]. Our department prefers to use the Pneumonia Severity Index in order to evaluate the severity of the disease. However, this score was not different between the two groups. The Pneumonia Score Index was calculated for patients in both groups and the Class range was between II-IV [16]. We repeated HINI test 7 days after admission and no patient negative in the initial test became positive.

In total, 60 patients were admitted in a four month period, of whom 33 were H1N1 positive (group A) and 27 negative (group B). The 33 H1N1 positive patients remained under quarantine and isolation, while the 27 negative patients were moved to the Department of Internal Medicine. Patients were monitored until discharge, with symptoms and signs recorded daily. Return to normal body temperature was defined as a temperature of less than 37°C for 1 day after withdrawal of antipyretic treatment [17]. The criteria for discharge were absence of hypoxemia, normal chest x-ray and temperature <37°C for 1 day without antipyretic treatment.

Upon admission procalcitonin (PCT) evaluation was performed in 51 patients (25 group A/26 group B), for nine patients this exam was not available due to lack in reagents in our laboratory. Also urine antigen for *Legionella *and *Streptococcus pneumoniae *was tested upon admission in all patients but they were negative. Upon admission sputum stain was given in 12/33 patients in group A and 15/27 in group B. The rest of the patients did not produce enough sputum quantity for staining or did not cooperate in giving sputum. Finally blood cultures were collected in 49/60 patients when the body temperature exceeded 38°C, but no results came positive.

## Statistical Analysis

Continuous variables were summarized as mean (±SD) or median (with interquartile ranges). For categorical variables, the percentages of patients in each category were calculated. Unpaired t test was used in normal distribution parameters comparing the mean values of the parameters of the two groups. A p value of less than 0.05 was considered to indicate statistical significance. Characteristics of the groups and clinical laboratory parameters were compared between the two groups (A, B). All analyses were carried out with the use of SPSS statistical software package (SPSS version 17.01; SPSS, Chicago, IL, USA).

## Clinical Characteristics of Patients

The majority of H1N1 positive patients (group A) were Caucasian male (23/33) with mean age 33.46 years and H1N1 negative patients (group B) were Caucasian male (17/27) with mean age 43.48 years. (Table 1.) Coexisting conditions were present in 25/33 patients in group A and 18/27 patients in group B (asthma: 24.2% vs. 25.9%, chronic obstructive disease: 6% vs. 0%, idiopathic pulmonary fibrosis: 3% vs. 0%, lymphoma: 15% vs. 3.7%, diabetes: 9% vs. 18.5%, coronary heart disease: 18% vs. 18.5%) as in previous reported studies [12,18]. (Table 1.) Only 2 patients in group A and 1 in group B had acute asthma exacerbation and none of the females in this study was pregnant. All female patients who were sexually active (8/33 group A) had negative pregnancy tests. All patients were treated with oseltamivir regimen (mean time 5.8 days in group A vs. 1.93 days in group B). In 7 patients of group A, additional treatment with azithromycin/moxifloxacin or ceftriaxone was added at the time of admission, due to local patchy shadowing on the chest x-Rays and fever >37.5°C. In 7/33 patients of group A and 10/27 of group B the second chest film was positive for pneumonia infiltrates. The patient's chest x-ray status upon admission is presented in table 2.

**Table 1:** Characteristics, underlying medical conditions and outcomes.

Characteristic	H1N1(+)	Range		(±SD)		H1N1(-)	Range	(±SD)		P Value
Age (years)										

Mean	33.46	14-65		(14.7)		43.48	16-86	(22.7)		0.057

Male/Female	23/10 (69.69%/30.3%)					17/10(62.96%/37%)				NS

Smoke	7/33 (21.2%)					7/27 (25.9%)				NS

BMI										

Mean	31.67	20-45		(9.15)		37.78	20-45	(8.0)		0.009

No,pts with obesity	18/33 (54.5%)					24/27 (88.8%)				

Coexistinsting conditions										

Asthma	8/33 (24.2%)					7/27 (25.9%)				NS

COPD	2/33 (6%)					0 (0%)				NS

IPF	1/33 (3%)					0 (0%)				NS

Lymphoma	5/33 (15%)					1/27 (3.7%)				NS

Diabetes	3/33 (9%)					5/27 (18.5%)				NS

Coronary Heart Disease	6/33 (18%)					5/27 (18.5%)				NS

Outcomes-days										

Duration of fever										

Mean	2.57	1-6		(1.0)		2.22	1-5	(1.0)		0.227

Days of Hospitalization										

Mean	6.11	2-18		(3.2)		5.85	2-12	(2.6)		0.750

Days under oseltamivir regimen										

Mean	5.8	5-18		(2.7)		1.93	1-5	(1.2)		NS

**Table 2:** Radiographic findings in the two groups, according to H1N1 status.

	H1N1(+)	H1N1(-)
Abnormalities on chest radiograph		

Local patchy shadowing	7/33 (21.2%)	10/27 (37.7%)

Ground-glass opacities	2/33 (6%)	7/27 (25.9%)

Interstitial abnormality	1/33 (3%)	0/27 (0%)

In 6 out of 12 patients of group A from whom sputum culture was received, a pathognomonic isolation (>10^6 ^CFU) of bacteria was achieved (3 *Streptococcus pneumoniae *species, 2 *Mycoplasma *species and 1 *Moraxella catarrhalis*). On the other hand from group B only in 8 out of 15 patients a pathognomonic isolation was achieved (5 *Streptococcus pneumoniae *species and 3 *Mycoplasma *species).

In group B, all patients were treated with double antibiotic regimen amoxicillin-clavulanic acid+azithromycin or ceftriaxone+moxifloxacin/ciprofloxacin (mean time of hospitalization was 5.85 days). Empiric antibiotic treatment was added upon admission based on elevated values of CRP, WBC and chest x-ray findings and since early antibiotic treatment prevents progression of the disease and these markers are known to be elevated in infection diseases [19-21]. Reiquelme et al [22] also initiated early antiviral and antibiotic treatment in order to prevent further progress and co-infection of the clinical status. Patients in our study were discharged when their chest radiograph became normal and their temperature <37°C for 1 day. Seven patients of group A and 6 patients of group B had severe hypoxemia upon admission (PO_2 _≤ 60 mmHg) on room air. (Table 1.) Hypoxemia was defined as decreased partial pressure of oxygen in blood ≤60 mmHg [23]. There was no difference between the two groups for smoking (A: n = 7/33 vs. B: n = 7/27). (Table 1.) Obesity was observed higher in patients from group B (mean 37.78) and a small number in group A (mean 31.67) (p value = 0.009). (Table 1.) According to WHO global database on Body Mass Index a patient is considered obese when BMI is ≥ 30 [24].

## Laboratory Findings

Leucopenia grade 1 (≥2,500 to <4,000/mm^3^) was observed in 2/5 patients diagnosed with lymphoma in group A. (Table 1.) In group B, only 1 patient had lymphoma and presented pneumonia. On admission, abnormal liver function (elevated levels of serum liver enzymes or bilirubin) was found in 4/33 patients in group A vs. 2/27 patients group B. (Table 1.) These patients were under no treatment. Moreover, there was no platelet abnormality in any patient of the two groups. C-reactive protein (CRP normal laboratory values <0.50 mg/dL) and white blood count (WBC) were elevated in group B in comparison to group A (mean 12.8 vs. 5.74).

Among the white blood count (WBC) subgroups there were no significant differences observed. The white blood count (WBC) returned to normal in 9/33 patients of group A and in 17/27 of group B between 6-12 days of hospitalization. C-reactive protein (CRP) returned to normal in 7/33 patients of group A and 27/27 of group B. Also in 4/33 patients in group A and 2/27 in group B elevated levels of serum liver enzymes or bilirubin returned to normal after 4-6 days. It should be mentioned that none of the patients received medications that affected their white blood count WBC (e.g. corticosteroids). (Table 3.)

**Table 3:** Clinical features of infection, according to H1N1 status: symptoms, signs and laboratory tests upon admission.

	H1N1(+)	Range	(±SD)	H1N1(-)	Range	(±SD)	P Value
Adverse events							

Abnormal liver function	4/33 (12%)			2/27 (7.4%)			NS

Nausea, Diarrhea	4.5%			1.5%			NS

Vomiting	1.4%			0.5%			NS

Hypoxemia	7/33 (21.2%)			6/27 (22.2%)			NS

CRP							

Mean	5.74	0.26-17.38	(4.75)	12.8	0.14-43.98	(11.56)	0.004

WBC							

Mean	7.114	3.340-10.950	(2172.160)	10,528	2.660-23.050	(5506.19)	0.004

Fever							

Mean	38.99	36.60-40	(0.79)	38.6	36.50-40.80	(1.01)	0.122

CR							

Mean	0.9	0.5-1.30	(0.18)	0.85	0.20-1.60	(0.25)	0.392

UR							

Mean	26.5	11-41	(7.9)	32.2	17-72	(11.8)	0.038

Spo_2_							

Mean	95.3	89-99	(2.7)	95.2	89-100	(2.4)	0.809

PO_2 _(FiO_2_21%)							

Mean	77.54	54-113	(14.6)	74,22	56-108	(13.0)	0.381

None of the patients had acute renal failure and none of the patients in the Unit of Infectious diseases had to be intubated and admitted to the ICU in comparison to other studies [7,25]. Also all patients were asked if they had vaccination for H1N1, but none of them had been vaccinated, even for seasonal influenza. There was no difference in mean saturation among the two groups. (Table 3.) Only 2/33 patients in group A with hypoxemia had to be hospitalized for 12 days and 18 days. The one of these 2 patients was recently diagnosed with idiopathic pulmonary fibrosis, while the other had no co- morbidities. No significant differences were observed for mean temperature, saturation and partial O_2 _between the two groups. (Table 3.)

## Discussion and Conclusions

We described a case series of sixty patients who were hospitalized in the Unit of Infectious diseases from 10^th ^August to 31^st ^December 2009 with flu-like symptoms and were tested with RT-PCR for H1N1 virus. Of these, 33 patients were positive for H1N1, while the remaining 27 were negative. The main differences between these two groups and corresponding clinical messages are summarized underneath.

In this case control study we included all patients with influenza symptoms admitted to the emergency flu department according to their attendance. Limitations of the study include that our data represent the experience of a single center, that procalcitonin test was given only in 51/60 patients and also Erythrocyte Sedimentation Rate (ESR) was sporadically collected during the follow up of the patients and so was not evaluated. Bacterial pneumoniae in association with influenza has been considered a important factor leading to poor patients outcomes in prior pandemics [26]. Even though none of the blood cultures were positive, we were unable to evaluate the effect of bacterial co-infection on patient outcomes, since blood cultures were obtained in only 17% of the study population (when fever ≥38°C) and workup for atypical pathogens was not performed. Although bacterial co-infection was not documented, the majority of the study population was treated with antibiotics. Prior publications failed to demonstrate any significant involvement of bacterial pathogens in hospitalized patients with 2009 H1N1 virus pneumonia [3,27-29]. During the initial evaluation in 4/27 patients of group B and 6/33 of group A an antibiotic treatment was prescribed by a General Practitioner and none of these patients had a sputum culture at that time. Furthermore, we did not receive cultivable sputum samples from all patients. We supposed that false negative culture in pneumonia patients is mainly due to mixed microbial flora or the natural colonization admixture of the upper airway. The subgroup of patients with pneumonia in both groups is so small that any statistical analysis is impossible and the power of the sample is quite small. Future studies are necessary to define the best treatment of 2009 H1N1 virus pneumonia and the role of combination antiviral therapy.

The lack of significant differences in the percentages of patients with hypoxemia between the two groups is probably due to the proximal number of patients with local patchy shadowing observed in group B and group A. (Table 2.)

Obesity is known to be associated with influenza A (H1N1) viral infection, but in this cohort we observed that in group B there was a larger number of obese patients in opposition to group A (88.8% vs. 54.5%) (p = 0.009). We were unable to explain the reason that the majority of H1N1 patients were not obese in our study as in previous reported studies [9,25]. Obesity is not a risk factor for poor outcomes in patients with seasonal influenza, but obesity has been suggested as a risk factor for poor outcomes in patients with 2009 H1N1 influenza infection in the USA [30].

In our case control study a large number patients suffering from lymphoma were observed, because these patients received chemotherapy regimen making them vulnerable to respiratory infections. Patients in group B had elevated C-reactive protein (mean 12.8 vs. 5.74) and white blood count WBC in comparison to group A (mean 10.528 vs. 7.114) suggesting a microbial infection already upon admission [19-21]. These elevated values (C-reactive protein and WBC) are known to be associated with bacterial infection and early antibiotic treatment prevents progression of the disease as reported in previous studies [19-21].

Symptoms from oseltamivir were mainly observed in group A (nausea 4.5% vs. 1.5%, diarrhea 4.5% vs. 1.5%, vomiting 1.4 vs. 0.5%) probably because of the larger dose and prolonged treatment with oseltamivir (5.8 vs. 1.93) as previously reported [18,31-33]. However, it was difficult to distinguish the pharmaceutical side effects of osetalmivir (tamiflu) from influenza symptoms in patients receiving antiviral treatment for less than 5 days [23]. Oseltamivir should be given until proof of negative RT-PCR result, since if a patient is positive, it prevents progression of the disease as shown in previous observational studies [17,34,35].

Moreover, mean duration of hospital stay was 5.85 in group B vs. 6.11 days in group A, because of the time needed for normalization of chest radiographs. Nevertheless, there were no significant differences between the two groups and the days of hospitalization were limited due to early oseltamivir for group A and antibiotic treatment for group B as previously explained.

Lastly the mean young age of the patients in both groups, and the small number of co-morbidities observed in our sample of patients, possibly were also responsible for having overall mild clinical course.

## In conclusion

All the patients in general, had a mild clinical course and none of the patients had to be admitted in the ICU.

## Competing interests

The authors declare that they have no competing interests.

## Authors' contributions

PZ collected and analyzed data, and wrote the manuscript; TK analyzed data and provided critical insights; KZ, NP and PS provided interpretation of data and finalized the version of the manuscript; EM collected and interpreted data and provided critical insight. All authors read and approved the final version of the manuscript.
